# Risk Perceptions and Psychological Effects During the Italian COVID-19 Emergency

**DOI:** 10.3389/fpsyg.2020.580053

**Published:** 2020-09-18

**Authors:** Tiziana Lanciano, Giusi Graziano, Antonietta Curci, Silvia Costadura, Alessia Monaco

**Affiliations:** ^1^Department of Education, Psychology, and Communication, University of Bari Aldo Moro, Bari, Italy; ^2^CORESEARCH, Center for Outcomes Research and Clinical Epidemiology, Pescara, Italy

**Keywords:** COVID-19, risk perception, risk-related variables, psychological effects, distress

## Abstract

The current study provides data about the immediate risk perceptions and psychological effects of the COVID-19 pandemic among Italian participants. A sample of 980 volunteers answered a web-based survey which aimed to investigate the many facets of risk perceptions connected to COVID-19 (health, work, institutional-economy, interpersonal and psychological), and risk-related variables such as perceived knowledge, news seeking, perceived control, perceived efficacy of containment measures, and affective states. Socio-demographic characteristics were also collected. Results showed that although levels of general concern are relatively high among Italians, risk perceptions are highest with regards to the institutional-economy and work, and lowest concerning health. COVID-19 has been also estimated to be the least likely cause of death. Cognitive and affective risk-related variables contributed to explain the several risk perception domains differently. COVID-19 perceived knowledge did not affect any risk perception while the perceived control decreased health risk likelihood. The other risk-related variables amplified risk perceptions: News seeking increased work and institutional-economy risk; perceived efficacy of containment measures increased almost all perceived risks; negative affective states of fear, anger and sadness increased health risk; anxiety increased health, interpersonal and psychological risks, and uncertainty increased work, institutional-economy, interpersonal and psychological risk perceptions. Finally, positive affective states increased health risk perception. Socio-psychological implications are discussed.

## Introduction

On 8th December 2019 the first case of Coronavirus 2019 (COVID-19) was identified in Wuhan (China), caused by the severe acute respiratory syndrome coronavirus 2 (SARS-CoV-2). The CoV-19 virus is believed to have originated from an infection probably obtained via zoonotic transmission starting at Wuhan’s seafood market. This event may be considered as the beginning of a global pandemic which, in only two months, has wreaked terrible damage all over the word. On 30th January 2020, the World Health Organization (WHO) declared a state of sanitary emergency. At the time of writing, Italy has been one of the most damaged countries with over 30.000 victims to date. On 21th February 2020, the first Italian case was registered in Codogno (Lombardy) and in under one month, the virus spread rapidly. In the first days of March, the Italian Government attempted to contain the spread of the virus and to prevent a collapse of the healthcare system by adopting drastic restrictions in the hardest hit regions called ‘Red Zones’. On 11th March 2020 a national lockdown was imposed to the whole country (Phase 1). To deal with the COVID-19 emergency, Italy moved into three phases, as follows:

– Phase 1 (11th March – 4th May) characterized by nationwide lockdown with compulsory restricted movement and imposed stay-at-home regulations, with the exception of specific circumstances.
– Phase 2 (4th May – 15th June) characterized by the relaxation of some restrictions; movement across regions was still prohibited, while traveling between municipalities was allowed only for proven reasons such as work, health and to visit relatives.
– Phase 3 (15th June – nowadays): access to indoor and outdoor places for entertaining activities has been permitted, with the requirement if retaining personal data of service users/clients for the following 14 days; face masks and social distancing have remained mandatory in enclosed public spaces, with the prohibition on gatherings.

The COVID-19 pandemic and the resulting containment measures have had devastating effects, upsetting and overwhelming people’s everyday lives and their perceptions of how dangerous the virus is. Several concurring aspects have come together to make the COVID-19 emergency a worldwide catastrophe without precedent: The impact of the virus has been global, it seemingly came out of nowhere and spread incredibly rapidly. It has so far claimed hundreds of thousands of lives and has resulted in confinement, enforced separation of families and friends and the restriction of movement and personal freedom. All these factors have contributed to great psychological distress and have forced people to look for new strategies to cope with and adjust to the emergency (Flesia et al., 2020; Liu et al., 2020; Losada-Baltar et al., 2020; Orrù et al., 2020; Polizzi et al., 2020; Wang et al., 2020). Wang et al. (2020) assessed the levels of psychological impact, anxiety, depression, and stress during the earliest phase of COVID-19 in China, finding a moderate-to-severe distress in more than half of their studied sample. The authors also intercepted factors associated with levels of well-being, suggesting possible psychological interventions to improve resilience and mental health during the pandemic. A recent review conducted by Brooks et al. (2020) found that predictors of psychological distress during quarantine are (a) longer duration of quarantine, (b) fear of infection, (c) frustration, (d) boredom, (e) inadequate supplies and inadequate information, (f) financial loss, and (g) stigma.

Among well-known COVID-19 psychological impacts, risk perception covers an important research area; the above-mentioned stressors drastically modified Italians’ risk perception. Considering the key role of behavioral and psychological reactions people have in facing pandemics, it is fundamental to assess how perceived risk is related to these. What risk did Italians actually perceive during the COVID-19 emergency? What worried them most? Were these worries restricted only to health? These are some of the research questions which have driven the current study.

### Risk Perception in Emergency Situations

According to Slovic and Peters (2006), in our modern world and 2.0 era, risk unfolds along two trajectories: a rational/cognitive risk referring to an analytic, systematic, deliberative and logical risk analysis and subsequent decision making; and an affective risk denoting an individual’s emotional and heuristic response to danger or threat. Several theories have remarked on the importance of emotion in risk perception and risk-taking behaviors, such as the model of affect-as-information (Schwarz and Clore, 1983, 2003), the risk as-feeling hypothesis (Loewenstein et al., 2001), and the affect heuristic (Slovic et al., 2007). Despite their differences, all these models feature the role of the affect and the emotional reactions playing in risk- judgment and decision making.

By investigating the perceived risk in the field of tourism for both man-made (e.g., terrorism) and natural disasters (e.g., tsunami or earthquake), Wolff et al. (2019) traced the many conceptualizations and measurement of risk perception. Some studies enquire into people’s worries and concerns, others assess people’s fear or nervousness, others measure the likelihood of events while others rate individuals’ riskiness, and so on. This great variability in risk perception measurement underlines the different facets of the construct, and the need for a clear and standardized operationalization.

Over the years, the vast majority of literature on risk perception has recommended the inclusion of cognitive, emotional and social dimensions which directly or indirectly characterize and influence people’s risk perception (Slovic, 1987, 1999; Slovic et al., 2000; Brug et al., 2004; Renn, 2006; Oh et al., 2015, 2020; van der Linden, 2015, 2017; Flesia et al., 2020). Using data collected during the 2015 Middle Eastern respiratory syndrome (MERS-CoV) outbreak in South Korea, empirical evidence suggested an association between risk perception and level of trust in social organizations (Yang and Cho, 2017). On the same data, Oh et al. (2020) found the role of social media in promoting preventive behaviors through fear and anger emotions which in turn affect people’s perception of risk. Oh et al. (2015) collected data on the 2009 H1N1 flu virus in South Korea and highlighted the role of cognitive (knowledge, controllability, and familiarity) and emotional (dread and immediacy) dimensions of risk characteristics on exposure to the media, and on personal- and societal-level risk perceptions.

The COVID-19 unexpected and deadly pandemic has led to a growing number of studies about its impact, and specifically, on risk perception with the aim to provide useful insights for subsequent risk communication strategies (Cori et al., 2020; Huynh, 2020; Taghrir et al., 2020). To briefly illustrate, in their study on COVID-19 risk perception in ten countries across Europe, Asia and America, Dryhurst et al. (2020) revealed that risk perception is significantly influenced by several predictors such as direct and indirect experience of the virus, personal and collective efficacy, personal knowledge, trust in the government, science, and medical professionals, and individual values and beliefs. Moreover, Kwok et al. (2020) investigated risk perception, anxiety level, sources to retrieve COVID-19 information, actual adoption and perceived efficacy of precautionary measures during the early phase of the COVID-19 epidemic in Hong Kong. The authors found that risk perception toward COVID-19 was high, and most people adopted self-protective measures and perceived them as effective. Additionally, de Bruin and Bennett (2020) assessed participants’ risks of COVID-19 infection and infection fatality and found that, despite some disagreements, participants who perceived greater risks were more likely to adopt protective practice, especially in the later stages of the COVID-19 spread. Lohiniva et al. (2020) presented weekly qualitative data collected by the Finnish Institute for Health and Welfare on COVID-19 risk perception in order to recommend appropriate risk communications. The narrative data was based on 116 email and social media posts and the findings were regrouped into five risk perception domains: catastrophic potential, probability of dying, reasons for exposure, belief of being in control of the situation, and trust toward authorities.

During the COVID-19 pandemic, people experienced several affective states beyond just fear and worry typically associated with risk perception, i.e., a sense of anxiety, anger, loneliness, frustration, confusion, inadequacy and uncertainty. By exploring the psychosocial outcomes due to quarantine because of exposure to severe acute respiratory syndrome (SARS), Robertson et al. (2004) found that quarantined health care workers experienced stigma, fear, and frustration. Facing up to the extreme uncertainty of COVID-19 may provoke devastating consequences (Chater, 2020; Lazzerini and Putoto, 2020). As a support, empirical evidence stressed the extent to which the intolerance of uncertainty is associated with anxiety and mood disorders (Boelen and Reijntjes, 2009), peaked levels of worry and rumination (Buhr and Dugas, 2006), and behaviors such as information seeking or monitoring and complying with recommendations (Rosen and Knäuper, 2009; Rosen et al., 2007). Flesia et al. (2020) recently revealed that the unpredictability and uncontrollability of the COVID-19 lockdown had a notable impact on predicting stress perceived during the emergency.

### The Current Study

The current study aimed to investigate COVID-19 risk perceptions in terms of likelihood and concern for consequences in all domains other than risk regarding health, i.e., risk connected to work, institutional-economy, interpersonal and psychological area (aim 1). Additionally, we explored the role of cognitive and affective risk-related variables to explain risk perceptions (aim 2). The idea behind this work is to investigate other risk domains beyond health emergency (i.e., risk of being infected, not healing and dying). We assessed risk perceptions and risk-related cognitive and affective variables using a survey that has been built by the authors and has been inspired by the literature.

Based on the most recent and ongoing studies on COVID-19, the pandemic has resulted in severely deleterious global outcomes: huge psychological strain (Mazza et al., 2020), traumatic impact from protracted social isolation and distance, shocking working aftermaths in terms of job loss and job search (Crayne, 2020), unemployment crisis (Blustein et al., 2020), economic crash across industries and countries (Fernandes, 2020). In their study, Chan et al. (2020) assessed risk perception of Health-Emergency Disaster Risk Management practices associated with COVID-19 in terms of perceived severity and infectivity, perceived knowledge to manage COVID-19, and perceived physical, mental, social, financial and global impact. All this empirical evidence about the COVID-19 emergency’s effects on different areas of people’s daily lives has led us to investigate the individual’s fear and concern for working, economy, interpersonal and psychological impact.

In line with previous studies on risk perception in emergency situations, we have also operationalized the risk-related cognitive dimension in terms of knowledge concerning COVID-19, news seeking, control and efficacy of containment measures. The risk-related affective dimension was operationalized in terms of affective states experienced during the COVID-19 emergency (i.e., anger, fear, worry, sadness, loneliness, anxiety, uncertainty, but also hope and trust). Considering that the main focus of the study is the subjective perception of risk, we decided to investigate the COVID risk-related cognitive and affective variables using a self-administered survey, therefore the cognitive and emotional dimensions here investigated are to be considered as ‘perceived’ and not objective (i.e., perceived knowledge, perceived control, and perceived efficacy).

For the cognitive dimensions, perceived knowledge refers to the perception that people have about how well they know a risk (Brug et al., 2004; Oh et al., 2015; Dryhurst et al., 2020). News seeking describes the information-seeking behavior typically associated with risk perception. Empirical evidence exists about the people’s need to seek risk information when making judgments and decisions on important issues. The two major motivations behind information-seeking behavior are increasing knowledge and reducing uncertainty. Risk perception is strictly related to information needs which in turn determines the subsequent search behavior (Neuwirth et al., 2000). Another important dimension that may affect the risk perception is controllability: If people perceive that they can control a risk, they will perceive the risk to be less severe (Oh et al., 2015). In the current study, we also assessed the perceived efficacy of political containment measures. This newly created measure is similar to several dimensions investigated in previous studies, such as trust (Dryhurst et al., 2020; Lohiniva et al., 2020), efficacy beliefs (De Zwart et al., 2009), belief of controllability of situation following the government’s restrictive measures (Lohiniva et al., 2020), and the efficacy of personal and collective actions in limiting the spread of coronavirus (Dryhurst et al., 2020).

## Materials and Methods

### Participants

A sample of nine hundred eighty Italians took part in a web-survey by using Google Forms platform. Table 1 shows all socio-demographic characteristics. The collection of data began on 14th April and ended on 19th April 2020, which was right in the middle of Phase 1 of the Italian COVID-19 lockdown. The sample was recruited on a voluntary basis, through word of mouth and via social media. All data were collected anonymously, and all participants provided informed research consent beforehand. The study was given ethical approval by the Ethics Committee of the Department of Education, Psychology, and Communication of the University of Bari Aldo Moro, and executed according to the Declaration of Helsinki (No. ET-20-01). Forms, material, and data are available on the Open Science Framework (OSF): https://osf.io/xdzkq/.

**TABLE 1:** Sample socio-demo characteristics.

	m ± sd	n (%)
**Age** (18–83 years)*	37.37 ± 13.74	
**Gender** (Women)		544 (55.5)
**Education**		
8th Grade		36 (3.7)
High school		413 (42.1)
University Degree		401 (40.9)
Post-degree		130 (13.3)
**Region of Residence**		
North		63 (6.4)
Center		37 (3.8)
South and Islands		880 (89.8)
**Employment**		
Student		213 (21.7)
Working student		39 (4)
Self-employed		124 (12.7)
Manager		46 (4.7)
Employee		412 (42)
Unemployed		59 (6)
Retired		4 (0.4)
Other		83 (8.5)
**Quarantine**		
No		14 (1.4)
Yes, I stay home		748 (76.3)
Yes, but I go to work		212 (21.6)
Yes, because I’ve been in contact with a COVID-19 positive		3 (0.3)
Yes, because I tested positive for COVID-19		3 (0.3)
**Marital status**		
Single		500 (51)
Married		370 (37.8)
Unmarried partner		59 (6)
Separated/Divorced		28 (2.9)
Widowed		6 (0.6)
Other		17 (1.7)
**Children at home** (No)		644 (65.7)
**N° housemates during quarantine**		
Alone		65 (6.6)
Two persons		180 (18.4)
3–5 persons		691 (70.5)
>5 persons		44 (4.5)
**Relatives living out** (Yes)		641 (65.3)
**Previous Pathologies** (No)		896 (91.4)

* *N* = 899; 81 participants incorrectly reported the birth date, so the data is missing.

### Measures

Participants completed a web survey containing several sections assessing socio-demographic characteristics, risk perceptions, and cognitive and affective processes to live and cope with COVID-19. See Supplementary Material for a list of the survey items.

#### Socio-Demographics Characteristics

This section assessed sample age, gender, education, region of residence, compliance with government regulations about quarantine, family status and cohabitation details (See Table 1).

#### Likelihood of COVID-19 Resolution

Participants answered two 11-point scale items (0 = not at all; 10 = very much) assessing the likelihood of the COVID-19 emergency being solved completely, and of people going back to their own everyday lives. Item scores were averaged into the Likelihood of Resolution index (α = 0.59).

#### Health Risk Perception – Concern and Likelihood

Participants answered nine 11-point scale items concerning health aspects (0 = not at all; 10 = very much). Items 1 to 3 assessed volunteers’ concerns for their own health, for health of their loved ones, and regarding a return to everyday life despite the risk of infection; Items 4 to 9 measured the likelihood estimation of contagion, death, and healing for themselves and others. Scores for items 1 to 3, and items 4 to 9 (items for healing were reversed) were averaged into indices of Health Risk Concern (α = 0.73) and Health Risk Likelihood (α = 0.71), respectively.

#### Mortality Risk

Participants answered six 11-point scale items (0 = not at all; 10 = very much) assessing the likelihood of dying from the following causes: (1) COVID-19, (2) Heart attack, (3) Stroke, (4) Cancer, (5) Dementia, and (6) Infection.

#### Work Risk Perception

Participants answered five 11-point scale items (0 = minimal influence; 10 = maximal influence) assessing the outcomes of COVID-19 in terms of (1) unemployment, (2) working management, (3) job prospects, (4) working self-efficacy, and (5) labor relations. Item scores were averaged into the Work Risk Perception index (α = 0.78).

#### Institutional-Economy Risk Perception

Participants answered four 11-points scale items (0 = minimal influence; 10 = maximal influence) assessing COVID-19 outcomes in terms of (1) financial crisis, (2) continuity of government, (3) EU relations, and (4) political landscape. Item scores were averaged into the Institutional-economy Risk Perception index (α = 0.85).

#### Interpersonal Risk Perception

Participants answered four 11-point scale items (0 = minimal influence; 10 = maximal influence) assessing the outcomes of COVID-19 in terms of (1) friendships, (2) family relationships, (3) love relationships, and (4) social cohesion. Item scores were averaged into the Interpersonal Risk Perception index (α = 0.82).

#### Psychological Risk Perception

Participants answered five 11-point scale items (0 = minimal influence; 10 = maximal influence) assessing COVID-19 outcomes in terms of (1) freedom, (2) self-actualization, (3) well-being, (4) isolation, and (5) thinking modalities. Item scores were averaged into the Psychological Risk Perception index (α = 0.86).

#### Perceived Knowledge

Participants answered an 11-point scale item (0 = not at all; 10 = very much) assessing the extent to which they consider themselves to be well- informed regarding COVID-19 (*‘How well-informed are you regarding COVID-19?’*).

#### COVID-19 Cause

Participants were asked what they thought was the most likely cause and origin of COVID-19 (see Table 2) (*‘In your opinion, what caused the virus?’*).

**TABLE 2:** Descriptive statistics for risk-related variables.

	m ± sd	n (%)
Perceived Knowledge	7.47 ± 1.46	
News Seeking		
Never		79 (8.1)
1 to 5 times		776 (79.2)
5 to 10 times		94 (9.6)
More than 10 times		31 (3.2)

Social networks		451 (46)
Chat		143 (14.6)
Institutional channels		903 (92.1)
Newspapers		430 (43.9)
Informal channels		154 (15.7)
Websites		430 (43.9)
Radio		95 (9.7%)
Causes		
Bat		206 (21)
Virus created in a lab		225 (23)
Chemical/Economic/Social war		41 (4.2)
Pre-existing virus evolution/Species leap		474 (48.4)
I don’t know/We will never know		26 (2.7)
Other		8 (0.8)
Perceived Control	5.98 ± 2.13	
Perceived Efficacy	7.92 ± 1.30	
Negative Affective States	4.91 ± 2.05	
Anxiety	4.85 ± 2.39	
Uncertainty	4.98 ± 2.44	
Positive Affective States	7.73 ± 2.02	

#### News Seeking

Participants were asked how much time they spent looking for news concerning the pandemic (1 = never; 2 = 1 to 5 times; 3 = 5 to 10 times; 4 = more than 10 times) (*‘How many times a day do you search for COVID-19 information?’*).

#### News Source

Participants answered questions about the sources mostly often used to search for COVID-19 information (social networks, chat, institutional channels, newspapers, informal channels, websites, radio, etc*.) (‘Choose the news sources you mostly used to keep up to date. You can choose multiple answers’*).

#### Perceived Control

Participants answered an 11-point scale item (0 = not at all; 10 = very much) to investigate perceived control concerning risk of infection (*‘How much do you think is it that you can control the likelihood of being infected?’*).

#### Perceived Efficacy of Containment Measures

Participants answered four 11-point scale items (0 = not at all; 10 = very much) to investigate (1) the efficacy of government containment measures, (2) the efficacy of compliance with government containment measures, (3) perceived safety by respecting government containment measures, and (4) efficacy of the contribution of each individual citizen during lockdown. Item scores were averaged into the Perceived Efficacy index (α = 0.78).

#### Affective States

Participants answered twenty 11-point scale items (0 = not at all; 10 = very much) assessing affective states during the COVID-19 emergency: (1) anger, (2) wrath, (3) fear, (4) anguish, (5) sadness, (6) depression, (7) loneliness, (8) nostalgia, (9) nervousness, (10) anxiety, (11) restlessness, (12) vulnerability, (13) impotence, (14) frustration, (15) inadequacy, (16) uncertainty, (17) confusion, (18) disorientation, (19) hope, and (20) trust. Scores for items 1 to 8 were averaged into the Negative Affective States index (α = 0.86); scores for items 9 to 12 were averaged into the Anxiety index (α = 0.81); scores for items 13 to 18 were averaged into the Uncertainty index (α = 0.88); scores for items 19 and 20 were averaged into the Positive Affective States index (α = 0.85).

### Statistical Methods

Descriptive statistics were calculated in order to examine socio-demographic characteristics and all the risk-related variables collected in the survey. Average scores with standard deviation and frequencies with percentages were used to summarize continuous and categorical variables, respectively. Two repeated-measure ANOVAs were run (1) to compare the measures of risk perception (health, work, institutional-economy, interpersonal, and psychological), and (2) to compare mortality risk for the different causes (COVID-19, heart attack, stroke, cancer, dementia, and infection). Results were graphically synthetized by boxplots. Pearson’s correlation coefficients were computed to explore the strength of the relation both among risk-perception measures and among risk-related variables. Separate multiple regression analyses were run to investigate the association of each perceived risk with the independent variables that were supposed to affect the outcome. The explanatory variables entered in each model were: Age, gender, education, employment, marital status, number of housemates during quarantine, relatives living far from home, previous pathologies, perceived knowledge, news seeking, perceived control, perceived efficacy, negative affective states, anxiety, uncertainty and positive affective states. The normal distribution of all outcomes was checked by calculating the values of skewness and kurtosis and graphically examining the model diagnostics. All variables included in these analyses were formally tested for collinearity on the basis of the variance inflation factor (VIF). Indicators of the relative importance of explanatory variables were also added in order to better understand the contribution of each of them both as direct and as combined with other variables in the model. Lindeman, Merenda and Gold’s (LMG) method (Lindeman et al., 1980) implemented in the R package “relaimpo” (Grömping, 2006) was adopted. LMG measures and their 95% bootstrap confidence intervals were plotted separately for each perceived risk. All results were considered statistically significant when *p*-value < 0.05. Statistical analyses were performed using SAS (version 9.4) and R software (release 3.5.2).

## Results

The main results are described in this section. Intra correlations among items composing each variable of interest, Variance Inflation Factor (VIF) values for the multicollinearity, and LMG measures are reported in Supplementary Material.

### Socio-Demographic Characteristics and Descriptive Analysis

As reported in Table 1, participants were balanced for gender (55.5% women), predominantly middle-aged (37 ± 13.74), and with a medium-high education level (42.1% high school degree; 40.9% University degree). Almost all volunteers making up the sample are resident in Southern Italy and islands (89.8%); 27.7% are not currently in employment (student and unemployed), whereas 63.4% are workers, with the remaining 8.9% belonging to other groups (e.g., retired). Almost the whole sample (98.5%) respected the lockdown and restrictive measures adopted by the government, either staying at home (76.3%) or mandatorily going to work; 51% of participants were single and 37.8% married. Slightly more than half of the sample (65.7%) lived in a house without children during the lockdown, with a large part of the sample (70.5%) spending the period of quarantine with 3 to 5 ‘housemates,’ including themselves. 65.3% of the sample had relatives living in other places, whilst 91.4% had no previous pathologies.

Table 2 shows descriptive statistics for all risk-related variables. Participants perceived themselves as quite knowledgeable about the COVID-19 pandemic, exhibited medium levels of control and attributed medium-high efficacy of government containment rules. Most of the sample followed the news up to 5 times a day through institutional and unofficial channels; and according to most of participants, the causes of COVID-19 were to be ascribed to the evolution of a pre-existing virus and species leap, a virus created in a lab, and bats. Finally, the most commonly experienced affective states were uncertainty, confusion and disorientation, but also trust and hope.

### Risk Perceptions

**FIGURE 1:**
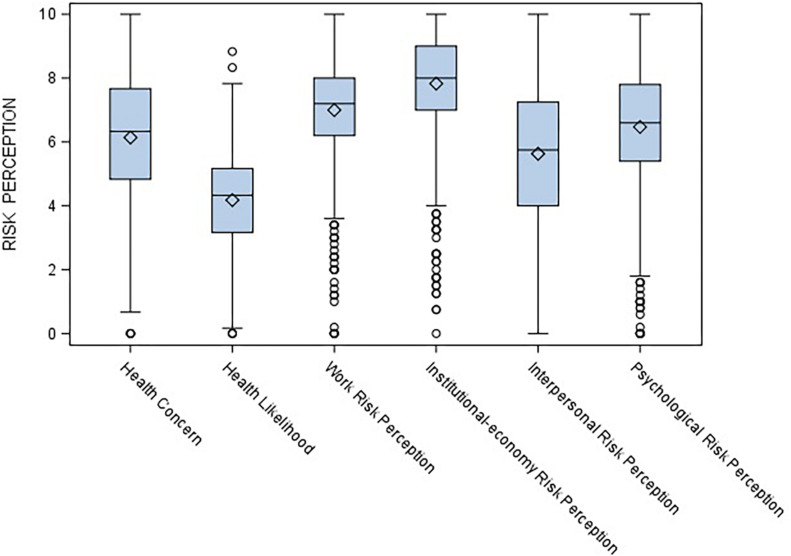
Repeated-measures ANOVA on risk perceptions.

**FIGURE 2:**
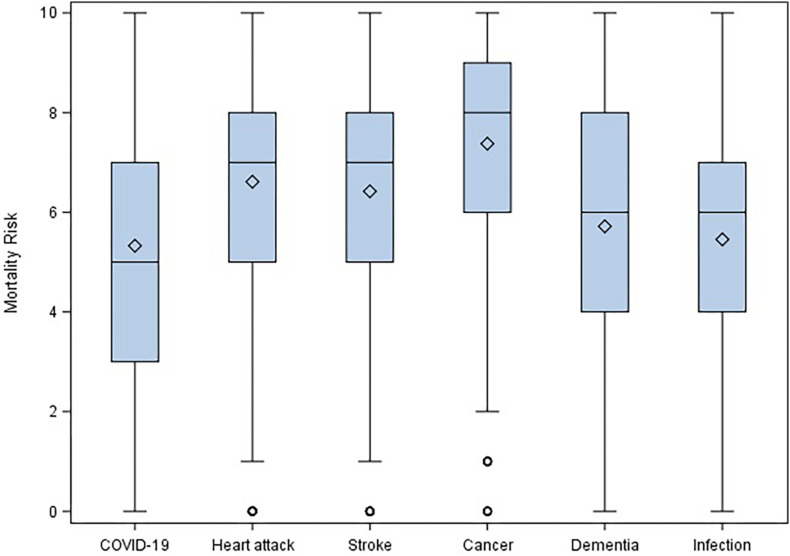
Repeated-measures ANOVA on mortality risk.

Table 3 shows means and standard deviations for the likelihood of resolution index, the six risk perception measures, and the mortality risk for the different causes (COVID-19, heart attack, stroke, cancer, dementia, and infection). Participants reported a low estimation of complete COVID-19 end and resolution, with the highest perceived risk was referred to institutional-economy and the lowest to health likelihood. Two repeated-measures ANOVAs were run to address the first aim of the study: The highest risks perceived by participants during the COVID-19 epidemic concerned institutional-economy and work, followed by psychological risk and, lastly, health (*F*_5_,_980_ = 430.29, *p* < 0.0001). Furthermore, cancer was evaluated as the most likely cause of death, while infections and COVID-19 as the least likely (*F*_5_,_980_ = 105.41, *p* < 0.0001). Figures 1 and 2 display the corresponding boxplots.

**TABLE 3:** Descriptive statistics for risk perception measures.

		Min-Max	m ± sd
	Likehood of Resolution	0–10	4.38 ± 2.24
Risk perceptions	*Health Concern*	0–10	6.14 ± 2.06
	*Health Likelihood*	0–8.83	4.18 ± 1.42
	*Work*	0–10	7.00 ± 1.64
	*Institutional-economy*	0–10	7.83 ± 1.76
	*Interpersonal*	0–10	5.63 ± 2.30
	*Psychological*	0–10	6.47 ± 1.97
Mortality risk	*COVID-19*	0–10	5.33 ± 2.41
	*Heart Attack*	0–10	6.61 ± 2.36
	*Stroke*	0–10	6.42 ± 2.41
	*Cancer*	0–10	7.38 ± 2.20
	*Dementia*	0–10	5.72 ± 2.57
	*Infection*	0–10	5.46 ± 2.52

### Correlations Among Risk Perception Measures and Risk-Related Variables

Table 4 shows Pearson’ correlations among risk perception measures, and risk-related variables, respectively. The likelihood of resolution is negatively associated with all risk perceptions, and the various risk perceptions are positively associated between each other. COVID-19 perceived knowledge is positively associated with news seeking, perceived control, perceived efficacy, and positive affective state, and negatively with anxiety and uncertainty. Searching for news is positively correlated to negative affective states, anxiety and uncertainty, whilst perceived efficacy of containment measures is positively linked to perceived control and emotions of hope and trust. Negative emotions appeared to be positively correlated to anxiety and uncertainty but also to positive feelings. Anxiety and uncertainty are strongly related to each other.

**TABLE 4:** Pearson’s Correlations among risk-perception measures and risk-related variables.

	Risk-perception measures						
	Health concern	Health likelihood	Work	Institutional-economy	Interpersonal	Psychological	
Likelihood of resolution	−0.11***	−0.16***	−0.09**	−0.14***	−0.13***	−0.17***	
Health concern		0.48***	0.27***	0.15***	0.37***	0.37***	
Health likelihood			0.16***	0.11***	0.25***	0.26***	
Work				0.49***	0.39***	0.52***	
Institutional-economy					0.24***	0.42***	
Interpersonal						0.59***	

	**Risk-related variables**						
	**News seeking**	**Perceived control**	**Perceived efficacy**	**Negative affective states**	**Anxiety**	**Uncertainty**	**Positive affective states**

Perceived knowledge	0.20***	0.10**	0.14***	–0.04	−0.08*	−0.08*	0.09**
News seeking		0.02	–0.06	0.13***	0.11***	0.10**	0.05
Perceived control			0.23***	0.03	0.02	0.01	0.06
Perceived efficacy				0.01	0.02	0.01	0.30***
Negative affective states					0.79***	0.72***	0.09**
Anxiety						0.75***	0.02
Uncertainty							0.05

* *p* < 0.05; ** *p* < 0.01; *** *p* < 0.001.

### The Role of Risk-Related Variables in Risk Perceptions

Table 5 reports results of the multiple regressions, run separately for the indices of likelihood of resolution and risk perceptions. Gender, education, relatives living out, perceived control, negative and positive affective states are found to significantly affect the likelihood of resolution. In particular, women, high-educated people and participants having relatives living out perceived a lower probability of a complete resolution (β = −0.80, *p* < 0.001; β = −0.05, *p* < 0.01; β = −0.41, *p* < 0.01, respectively). Gender female is also associated with the health concern (β = 0.57, *p* < 0.001), institutional-economy (β = 0.34, *p* < 0.01), and psychological (β = 0.36, *p* < 0.01) risk perceptions. The presence of previous pathologies shows a positive contribution to health likelihood (β = 0.58, *p* < 0.001) and a negative contribution to work (β = −0.50, *p* < 0.01) and institutional-economy (β = −0.04, *p* < 0.05) risk perception.

**TABLE 5:** Multiple regression analyses.

Predictors	Likelihood of resolution (*R*^2^=0.15; F_(__21_,_898__)_=7.52***)		Health concern (*R*^2^=0.40; F_(__21_,_898__)_=27.81***)		Health likelihood (*R*^2^=0.18; F_(__21_,_898__)_=9.04***)		Work risk (*R*^2^=0.13; F_(__21_,_898__)_=6.34***)		Institutional-economy risk (*R*^2^=0.10; F_(__21_,_898__)_=4.84***)		Interpersonal risk (*R*^2^=0.16; F_(__21_,_898__)_=8.07***)		Psychological risk (*R*^2^=0.24; F_(__21_,_898__)_=13.21***)	
							
	β	*t*	β	*t*	β	*t*	β	*t*	β	*t*	β	*t*	β	*t*
Age														
Gender (F)	−0.80	−5.10***	0.57	4.75***					0.34	2.71**			0.36	2.77**
Education	−0.05	−2.86**	−0.03	−2.61**	−0.03	−2.61**			0.03	2.01*				
Employment														
Marital status														
N° housemates														
Relatives living out (Yes)	−0.41	−2.72**	0.48	4.09***	0.28	3.01**					0.43	2.80**	0.30	2.38*
Previous Pathologies (Yes)					0.58	3.60***	−0.50	−2.61**	−0.04	−2.09*				
Perceived Knowledge														
News Seeking							0.22	2.04*	0.26	2.23*				
Perceived Control	0.15	4.41***			−0.09	−4.18***								
Perceived Efficacy			0.14	3.12**			0.17	3.93***	0.22	4.50***	0.17	2.85**		
Negative Affective States	0.16	2.65**	0.25	5.22***	0.08	2.09*								
Anxiety			0.25	6.10***	0.15	4.34***					0.11	2.02*	0.14	3.28**
Uncertainty							0.11	3.32**	0.12	3.24**	0.15	3.20**	0.18	4.71***
Positive Affective States	0.19	5.14***	0.10	3.67***										

* *p* < 0.05; ** *p* < 0.01;*** *p* < 0.001.

The more people seek news the more they perceive the work and institutional-economy risk (β = 0.22, *p* < 0.05; β = 0.26, *p* < 0.05, respectively). The perceived control shows a negative contribution to health likelihood (β = −0.09, *p* < 0.001), while the perceived efficacy of the containment measures shows a positive contribution to health concern (β = 0.14, *p* < 0.01), work (β = 0.17, *p* < 0.001), institutional-economy (β = 0.22, *p* < 0.001), and interpersonal (β = 0.17, *p* < 0.01) risk perceptions. As regards the affective risk-related variables, they are found to positively contribute to the level of risk perception.

**FIGURE 3:**
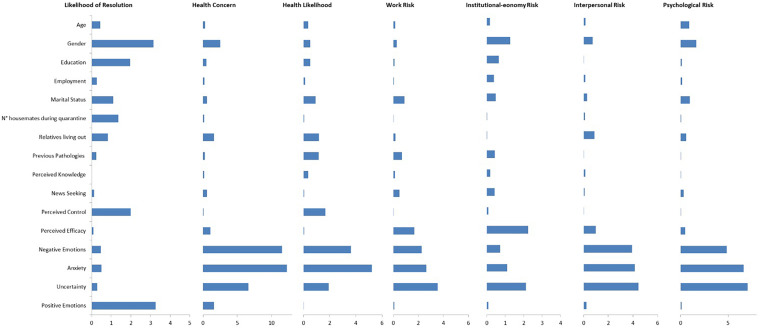
LMG measures.

Figure 3 synthesizes the LMG measures, separately for each outcome. The contribution of socio-demographic variables, perceived control and positive affective state to the *R*^2^ of models is relevant only for likelihood of resolution. Together they explain more than 10% of variance. Perceived efficacy about the containment measures emerges as important variable for the institutional-economy risk [2.23%, 95% CI (0.79-4.26)]. A substantial proportion of variance of health concern, health likelihood, interpersonal risk and psychological risk is explained by negative affective state (fear, anger, sadness) and anxiety. Uncertainty is the first relevant variable for work risk (3.54%, 95% CI [2.05-5.69]), interpersonal risk (4.46%, 95% CI [2.89-6.59]) and psychological risk (7.06%, 95% CI [4.99-9.53]).

## Discussion

The COVID-19 pandemic has indelibly and irreversibly changed the whole world. Nothing (or almost nothing) will ever be the same as it once was. In Italy, the devastating effects of the lockdown have had an impact on several domains: The collapsing health system, a deep and difficult-to-solve economy-work crisis, high levels of distress and so on. The short and long-term effect of COVID-19 also made its mark on how people have perceived and represented the ongoing events and future scenarios, including risk perception. The current study provides data regarding COVID-19 immediate risk perceptions in terms of likelihood and concern in all risk domains beyond health, i.e., risk connected to work, institutional-economy, interpersonal and psychological areas. Additionally, the study aimed to investigate the role of socio-demographic characteristics and cognitive and affective risk-related variables to impact perceived risks in response to the COVID-19 epidemic.

To answer our first aim, current results showed that the risk perception of being infected by the virus or of dying from COVID-19 was not the highest perceived risk, with participants instead showing high levels of concern for institutional-economy and work in the future, followed by psychological worry. This finding clearly suggests how, after the health emergency, it was and remains today important to manage the social and psychological emergency. People did not estimate a high probability of becoming infected or dying, instead they perceived a great deal of concern and fear for the future and for economic and social consequences of the pandemic. Crucially, this worry will spread over time and will not disappear with the clinical extinction of the virus. During the COVID-19 lockdown, individuals were forced to listen to fearful messages such as the daily bulletin of Civil Protection Agency about the dramatic increase of contagions and deaths, or the evening live television broadcasts with the Prime Minister’s COVID-related announcements. These messages may have been processed in a more rational or more emotional way. People generally process information in two different ways: A systematic vs. heuristic processing (the dual-process model of communication). According to the well-known Heuristic-Systematic Model (HSM; Chaiken, 1980), when people are asked to form a judgment or make a decision, they may systematically and analytically process any available information, or adopt cognitive shortcuts and heuristic processing which *‘may involve the use of relatively general rules (scripts, schemata) developed by individuals through their past experiences’* (Chaiken, 1980, p. 753). The high levels of risk perceptions beyond the health emergency suggest that Italian citizens dealt with the COVID-related news and experienced lockdown and restrictive measures in an ‘emotional’ way (rather than rational), by feeling peaks of fear and concern. Probably, this fear will linger through time. Fearful communication is based on fear, an unpleasant and evolutionary emotion that responds to the function of protecting humans from life-threatening situations (Williams, 2012). Anchoring to the Appraisal Tendency Framework (ATF; Lerner and Keltner, 2000), fear that people experienced during the COVID-19 emergency affected their perceptions of risk; people in the fear condition are prone to perceive a greater risk on the basis of a sense of uncertainty, vulnerability and lack of control over the situation (Lerner and Keltner, 2000, 2001; Lerner et al., 2003).

As regards socio-demographic characteristics, gender, education, relatives living far from home, and previous pathologies appear to significantly contribute to risk perceptions.

Specifically, women reported higher risk perceptions than men (except for work and interpersonal risk), and highly educated female participants are those who estimated the lowest probability of the COVID-19 emergency being solved completely, and of people going back to a normal everyday life. Recent COVID data showed that women are more concerned about COVID-19 than men (Gerhold, 2020). Moreover, high education seemed to influence a high institutional-economy risk perception, and a low health risk perception in terms of concern and likelihood. It is as if a high level of education protected people from a (possible) irrational fear of being infected or dying, but at the same time permitted them to realize the devastating economic, political and institutional scenario that was coming up. Additionally, having loved ones living far from home increased health, interpersonal and psychological risk perceptions and, coherently, decreased the estimated probability of resolution. Previous pathologies resulted as being associated with a high health risk but low work and institutional-economy risk. Our data roughly overlap with the results obtained by Bish and Michie (2010) in their review concerning the demographic and attitudinal predictors of behaviors during a pandemic. The authors concluded that being older, female and more educated is associated with a higher probability to engage in protective and preventing behaviors, and this link is mediated by several attitudes, such as higher levels of perceived susceptibility to the disease.

Furthermore, as regards the second aim of the study, results showed how risk-related cognitive and affective variables differently impact the various risk perceptions, by confirming that risk perceptions are distinct and need to be investigated independently between each others.

In detail, COVID-19 perceived knowledge does not affect any single risk perception and this seems to be an unexpected finding. By adopting the above-cited HSM framework to understand how individuals process and respond to fearful messages, Averbeck et al. (2011), investigated the role of prior knowledge in systematic vs. heuristic processing fear appeals. The authors found that prior knowledge gives rise to systematic processing by attending to context-relevant information, whilst a lack of prior knowledge leads to heuristically processing fearful messages by resulting in greater fear arousal. Our contradictory result concerning the no-role of knowledge on risk perception may be explained by considering that messages, information and appeals leaked by our Government and news media were not only fearfully charged, but also full of uncertainty and eliciting confusion. Hence, it was difficult to be truly informed on such a new and unexpected topic, like COVID-19 – the so-called ‘invisible enemy’ – which continues to divide the scientific community as it searches for an answer to the crisis.

Instead, frequent searching for information on COVID-19 increased fear and concern for the institutional-economy and working future. The mass media contributed greatly to risk perceptions, especially for those risk situations in which individuals do not have first-hand experience or adequate and sufficient knowledge, so that they seek, in the mass media, information necessary to resolve uncertainty and confusion. In cases as such one, the mass media serves as a ‘social amplification’ since they allow people both to learn about the risk message and interpret it (Social Amplification of Risk Framework; Kasperson et al., 1988), hence the mass media amplify or weaken the public’s perception of risk (Chong and Choy, 2018; Ali et al., 2019). Our finding might be understood by considering that the real risk and threat perceived by people was the limited sense of predictability and controllability assigned to the whole situation, rather than the virus *per se* (Flesia et al., 2020). This role played by the mass media has been traced in several health communication studies such as that on Avian flu in Hong Kong and the United States (Fung et al., 2011), 2009 H1N1 flu virus (Oh et al., 2015), bovine spongiform encephalopathy (Paek et al., 2016), or 2015 Middle East Respiratory Syndrome coronavirus in South Korea (Oh et al., 2020).

Instead, high levels of perceived control increased people’s estimate of a solution to COVID-19, and reduced the perceived likelihood of health risk for themselves and their loved ones. It has been well established that perception of control plays a crucial role on how people formulate judgments and make decisions about risk, by leading people to underestimate risks under their control (Thompson et al., 1998; Beisswingert et al., 2015). By differentiating between risk control (‘command over the result’) and volition (‘command over the risk exposure’), Nordgren et al. (2007) found that control resulted in a decreasing perceived risk, while volition resulted in increasing perceived risk. Perceived control represents a construct strictly related to a range of psychological variables and widely mentioned in several motivation theories, such as – among many – the Self-Determination Theory (SDT; Ryan and Deci, 2000) which encompasses three human needs underlying intrinsic motivation: Autonomy, competence and relatedness. The first claims that developing a sense of autonomy and control over situations is fundamental for an individual to be able to self-regulate, maintain and internalize recommended behaviors, such as respecting rules, complying legal measures, or adhering to medical prescriptions (Williams et al., 1998; Ryan et al., 2008). In this vein, people with high levels of perceived control concerning the probability of being infected exhibited lower health risk perception.

Interestingly, perceiving as effective the government’s containment measures and the protective behaviors increased health, work, institutional-economy, and interpersonal risk perceptions, but not the health risk likelihood. This seemingly strange result – *the higher the perception of efficacy, the higher the perception of risk* – might be explained by invoking the fear appraisal processes. People perceive lockdown measures and compliance conducts as effective, this perception of effectiveness, in turn, endorses the existence of an objective risk perceived as a threat from which individuals should protect themselves. This objective risk, in turn, sustains fear and subjective risk felt by people. It is as if the link between perceived efficacy and risk perception is circular rather than linear. The perceived efficacy seems to be unrelated with the health risk likelihood and health risk severity (death likelihood), while it seems to affect fear and concern for health and not only; therefore, in other words it seems to affect the threat appraisal. The more people perceive containment measures and individual/collective compliance behaviors as effective, safe and relevant, the more fear and concern for health and for the working, institutional-economy and social future increases. In the recent study about risk perceptions of COVID-19 around the world, Dryhurst et al. (2020) found a partially similar result showing a positive correlation of risk perception with personal efficacy (‘*To what extent do you feel that the personal actions you are taking to try to limit the spread of coronavirus make a difference?’*, p. 4) but a negative correlation with collective efficacy (*‘To what extent do you feel the actions that your country is taking to limit the spread of coronavirus make a difference?’*, p. 4). This our result may be explained by mentioning the Protection Motivation Theory (PMT; Rogers, 1975, 1983) according to which people must first believe that a threat be directed at them (threat appraisal) and then evaluate to adopt preventive behaviors (coping appraisal). In this vein, the belief of efficacy about the restrictive measures, the protective compliance behaviors, and the contribution of the individual citizen would have increased the threat appraisal. PMT posits that response efficacy (i.e., people’s believe that protective actions are effective) and self-efficacy (i.e., people’s believe to be able to adopt protective behaviors) are two predictors of protection motivation (Rogers, 1983).

Furthermore, both negative and positive affective states predicted the likelihood of resolution. The risk-related affective variables in terms of fear, anger, sadness, anxiety and positive emotions have mainly influenced the perception of health. Anxious affective states amplified interpersonal and psychological risk perception, whilst uncertainty enhanced perceived risks for work, institutional-economy, and psycho-social area. Witte and Allen’s meta-analysis (Witte and Allen, 2000) on fear appeal studies concluded that fearful messages produce a peak of perceived severity and vulnerability, and result as being greatly persuasive in encouraging people to adopt desired behaviors. In this vein, the strategic and communicative decisions made by our Government called upon an affective response of fear for facing the COVID-19 risk, with the aim to increase risk perception and, consequently, motivate people to adopt the recommended behaviors (Dillard and Anderson, 2004; Averbeck et al., 2011). The Italian hashtag #iorestoacasa (*I stay at home*) perfectly embodied the extraordinary restrictive measures taken by the Prime Minister (DPCM), becoming a viral trend on social media and flash mobs across all of Italy. Italians, in response, overall complied with the government’s restrictive measures, but this heightened their COVID-19 risk perception. Although the daily ‘death’ bulletin of the Civil Protection Agency is a fact, future studies should accurately investigate the risky communication adopted by politicians in order to be able to express more about a link between risk communication and risk perception. To sum up, one can speculate that the restrictive measures adopted by the Italian government – albeit considered effective – accompanied by a communicative style that oscillated between fear and uncertainty increased the individual’s risk perception. It would seem that fearful and uncertain communication did not help people, but just served to frighten them further.

The risk-related cognitive variables of news seeking, perceived control and efficacy and the affective variables of fear, anxiety, and uncertainty seem thus to have influenced risk perceptions.

The study has some limitations. First, it is a cross-sectional study, so direct causal inferences about the relationship between risk-related variables and risk perception measures need to be made cautiously. Second, the sample is not representative of all Italian regions and of course we know well that the situation was much more serious in the country’s northern regions due to the far higher number of infections. However, we assumed that the risk perception and concern were equally distributed throughout the Italian country: As the Italian government extended the lockdown, millions of citizens living in the North regions (above of Lombardy and Veneto) fled south on the last departing trains and buses. Third, we did not adopt standardized measures to assess psychological distress or risk-taking style but we instead used a personalized battery aimed to assess the constructs of interest. Despite these limitations, however, the study offers food for thought in order to better understand the complexity of the psychological experiences by one of the countries which has been hardest hit by the virus. It will be vital for politicians that decisions made from above are understood in the light of all psychological processes involved and here analyzed. At the time of writing, the COVID-19 pandemic is likely to bring a second wave of socio-psychological emergency. The individuals’ wellbeing cannot be reduced to a merely medical wellbeing.

## Data Availability Statement

The datasets presented in this study can be found in online repositories. The names of the repository/repositories and accession number(s) can be found in the article/Supplementary Material.

## Ethics Statement

The studies involving human participants were reviewed and approved by Ethics Committee of the Department of Education, Psychology, and Communication of the University of Bari Aldo Moro (No. ET-20-01). The patients/participants provided their written informed consent to participate in this study.

## Author Contributions

TL, SC, and AM conceptualized and designed the work. TL, GG, SC, and AM acquired and analyzed the data. TL and GG interpretated the data. TL, GG, and AC drafted the work. TL, GG, AC, SC, and AM performed revising and providing the final approval of the work. All authors contributed to the article and approved the submitted version.

## Conflict of Interest

The authors declare that the research was conducted in the absence of any commercial or financial relationships that could be construed as a potential conflict of interest.
