# Supplementary material for: Risk Perceptions and Psychological Effects During the Italian COVID-19 Emergency
Source: Front Psychol. 2020 Sep 18;11:580053. doi: 10.3389/fpsyg.2020.580053 (PMC7533588; doi:10.3389/fpsyg.2020.580053)
Supplement: Supplementary file 1 [file Table_1.docx]

Supplementary Material

**Risk Perceptions and Psychological Effects during the Italian COVID-19 Emergency**

Tiziana Lanciano^1^*, Giusi Graziano^2^, Antonietta Curci^1^, Silvia Costadura^1^, Alessia Monaco^1^

^1^Department of Education, Psychology, and Communication, University of Bari Aldo Moro, Bari, Italy

^2^CORESEARCH, Center for Outcomes Research and Clinical Epidemiology, Pescara, Italy

Correspondence to: Tiziana Lanciano, Department of Education, Psychology, and Communication, University of Bari Aldo Moro, Bari, Italy E-mail: tiziana.lanciano@uniba.it

**Table of Contents**

Table S1. Intracorrelations among Likelihood of COVID-19 Resolution items

Table S2. Intracorrelations among Health Concern items

Table S3. Intracorrelations among Health Likelihood items

Table S4. Intracorrelations among Work Risk Perception items

Table S5. Intracorrelations among Institutional-economy Risk Perception items

Table S6. Intracorrelations among Interpersonal Risk Perception items

Table S7*.* Intracorrelations among Psychological Risk Perception items

Table S8*.* Intracorrelations among Perceived Efficacy items

Table S9. Intracorrelations among Affective States items

Table S10. Variance Inflation Factor (VIF) values for the multicollinearity

Table S11. LMG measures with 95% bootstrap confidence interval

**Table S1.** Intracorrelations among Likelihood of COVID-19 Resolution items

|  | 2. How likely do you think it is that after the containment measures you will immediately return to your previous everyday life? *(returning to daily life with no chance of being infected)* |
| --- | --- |
| 1. How likely do you think it is that the COVID-19 emergency will be resolved completely? *(returning to daily life with no chance of being infected)* | .42* |

**p < .001*

**Table S2.** Intracorrelations among Health Concern items

|  | 2. How worried/concerned are you about the lives of your loved ones? | 3. How worried/concerned are you at the thought of going back to daily life because of the risk of infection? |
| --- | --- | --- |
| 1. How worried/concerned are you about your life? | .53* | .52* |
| 2. How worried/concerned are you about the lives of your loved ones? |  | .39* |

**p < .001*

**Table S3.** Intracorrelations among Health Likelihood items

|  | 2. How likely do you think it is that you might die of COVID-19? | 3. How likely do you think it is that you might recover from COVID-19? *(considering your current health conditions)* | 4. How likely do you think it is that your loved ones might become infected with COVID-19? | 5. How likely do you think it is that your loved ones might die of COVID-19? | 6. How likely do you think it is that your loved ones might recover from COVID-19? *(considering their current health conditions)* |
| --- | --- | --- | --- | --- | --- |
| 1. How likely do you think it is that you might become infected with COVID-19? | .52** | .11* | .56** | .33** | -.05 |
| 2. How likely do you think it is that you might die of COVID-19? |  | .43** | .41** | .41** | .13* |
| 3. How likely do you think it is that you might recover from COVID-19? *(considering your current health conditions)* |  |  | .01 | .06 | .45** |
| 4. How likely do you think it is that your loved ones might become infected with COVID-19? |  |  |  | .60** | -.02 |
| 5. How likely do you think it is that your loved ones might die of COVID-19? |  |  |  |  | .22** |

**p< .01; **p < .001*

**Table S4.** Intracorrelations among Work Risk Perception items

| How much do you think COVID-19 might affect the following domains during the next year? | 2. Working management | 3. Job prospects | 4. Working self-efficacy | 5. Labour relations |
| --- | --- | --- | --- | --- |
| 1. Unemployment | .49* | .60* | .34* | .25* |
| 2. Working management |  | .50* | .37* | .43* |
| 3. Job prospects |  |  | .42* | .33* |
| 4. Working self-efficacy |  |  |  | .45* |

**p< .001*

**Table S5.** Intracorrelations among Institutional-economy Risk Perception items

| How much do you think COVID-19 might affect the following domains in the next year? | 2. Continuity of government | 3. EU relations | 4. Political landscape |
| --- | --- | --- | --- |
| 1. Financial crisis | .45* | .48* | .50* |
| 2. Continuity of government |  | .60* | .66* |
| 3. EU relations |  |  | .82* |

**p< .001*

**Table S6.** Intracorrelations among Interpersonal Risk Perception items

| How much do you think COVID-19 might affect the following domains during the next year? | 2. Family relationships | 3. Love relationships | 4. Social cohesion |
| --- | --- | --- | --- |
| 1. Friendship | .66* | .49* | .61* |
| 2. Family relationships |  | .61* | .46* |
| 3. Love relationships |  |  | .47* |

**p< .001*

**Table S7***.* Intracorrelations among Psychological Risk Perception items

| How much do you think COVID-19 might affect the following domains during the next year? | 2. Self-actualization | 3. Well-being | 4. Isolation | 5. Thinking modalities |
| --- | --- | --- | --- | --- |
| 1. Freedom | .61* | .67* | .53* | .46* |
| 2. Self-actualization |  | .69* | .52* | .44* |
| 3. Well-being |  |  | .60* | .51* |
| 4. Isolation |  |  |  | .45* |

**p< .001*

**Table S8***.* Intracorrelations among Perceived Efficacy items

|  | 2. To what extent do you believe that complying with the containment measures decreases the likelihood of being infected? | 3. To what extent does complying with the containment measures make you feel safe? | 4. To what extent do you believe that the behavior of each individual citizen might influence the spread of the virus? |
| --- | --- | --- | --- |
| 1. How effective do you believe the government’s measures are in containing the spread of COVID-19? | .55* | .51* | .29* |
| 2. To what extent does complying with the containment measures decrease the likelihood of being infected? |  | .66* | .46* |
| 3. To what extent does complying with the containment measures make you feel safe? |  |  | .35* |

**p< .001*

**Table S9.** Intracorrelations among Affective States items

| 1. How much have you felt or are you feeling the following emotions? | *Wrath* | *Fear* | *Anguish* | *Sadness* | *Depression* | *Loneliness* | *Nostalgia* | *Anxiety* | *Restlessness* | *Vulnerability* | *Frustration* | *Inadequacy* | *Uncertainty* | *Confusion* | *Disorientation* | *Trust* |
| --- | --- | --- | --- | --- | --- | --- | --- | --- | --- | --- | --- | --- | --- | --- | --- | --- |
| *Anger* | .69^*^ | .45^*^ | .48^*^ | .46^*^ | .35^*^ | .25^*^ | .35^*^ |  |  |  |  |  |  |  |  |  |
| *Wrath* |  | .33^*^ | .43^*^ | .40^*^ | .43^*^ | .28^*^ | .25^*^ |  |  |  |  |  |  |  |  |  |
| *Fear* |  |  | .65^*^ | .51^*^ | .34^*^ | .25^*^ | .31^*^ |  |  |  |  |  |  |  |  |  |
| *Anguish* |  |  |  | .65^*^ | .53^*^ | .40^*^ | .44^*^ |  |  |  |  |  |  |  |  |  |
| *Sadness* |  |  |  |  | .57^*^ | .49^*^ | .55^*^ |  |  |  |  |  |  |  |  |  |
| *Depression* |  |  |  |  |  | .54^*^ | .37^*^ |  |  |  |  |  |  |  |  |  |
| *Loneliness* |  |  |  |  |  |  | .42^*^ |  |  |  |  |  |  |  |  |  |
| *Nervousness* |  |  |  |  |  |  |  | .58^*^ | .55^*^ | .39^*^ |  |  |  |  |  |  |
| *Anxiety* |  |  |  |  |  |  |  |  | .60^*^ | .51^*^ |  |  |  |  |  |  |
| *Restlessness* |  |  |  |  |  |  |  |  |  | .50^*^ |  |  |  |  |  |  |
| *Impotence* |  |  |  |  |  |  |  |  |  |  | .47^*^ | .50^*^ | .54^*^ | .49^*^ | .51^*^ |  |
| *Frustration* |  |  |  |  |  |  |  |  |  |  |  | .53^*^ | .48^*^ | .51^*^ | .57^*^ |  |
| *Inadequacy* |  |  |  |  |  |  |  |  |  |  |  |  | .50^*^ | .56^*^ | .61^*^ |  |
| *Uncertainty* |  |  |  |  |  |  |  |  |  |  |  |  |  | .60^*^ | .54^*^ |  |
| *Confusion* |  |  |  |  |  |  |  |  |  |  |  |  |  |  | .79^*^ |  |
| *Hope* |  |  |  |  |  |  |  |  |  |  |  |  |  |  |  | .73^*^ |

**p< .001*

**Table S10.**Variance Inflation Factor (VIF) values for the multicollinearity

| **Variable** | **VIF** |
| --- | --- |
| Age | 2.00632 |
| Gender | 1.23944 |
| Education | 1.12222 |
| Employment | 1.42289 |
| Marital status | 1.33661 |
| N° of housemates during quarantine | 1.06494 |
| Relatives living far from home | 1.04157 |
| Previous Pathologies | 1.09884 |
| Perceived Knowledge | 1.11965 |
| News Seeking | 1.18339 |
| Perceived Control | 1.07065 |
| Perceived Efficacy | 1.21891 |
| Negative Affective States | 3.25096 |
| Anxiety | 3.34845 |
| Uncertainty | 2.64123 |
| Positive Affective States | 1.15925 |

**Table S11.**LMG measures with 95% bootstrap confidence interval

|  | Likelihood of  Resolution | Health  Concern | Health  Likelihood | Work  Risk Perception | Institutional-economy  Risk Perception | Interpersonal  Risk Perception | Psychological  Risk Perception |
| --- | --- | --- | --- | --- | --- | --- | --- |
| Age | .44  (.15-1.21) | .29  (.20-.79) | .37  (.10-1.18) | .15  (.07-.59) | .17  (.08-.73) | .15  (.08-.62) | .90  (.36-1.88) |
| Gender | 3.14  (1.41-5.46) | 2.51  (1.3-4.06) | .52  (.11-1.59) | .28  (.07-1.21) | 1.27  (.36-3.08) | .73  (.15-1.96) | 1.65  (.65-3.3) |
| Education | 1.95  (.69-3.82) | .50  (.07-1.46) | .52  (.04-1.67) | .09  (.01-.8) | .65  (.05-1.96) | .02  (.01-.56) | .15  (.02-.91) |
| Employment | .27  (.11-1.28) | .23  (.10-.91) | .13  (.06-.95) | .04  (.04-.77) | .39  (.09-1.66) | .13  (.04-.89) | .16  (.12-.78) |
| Marital status | 1.10  (.60-2.94) | .55  (.3-1.72) | .93  (.47-2.77) | .89  (.4-3) | .48  (.27-2.22) | .27  (.18-1.59) | .97  (.55-2.63) |
| N° housemates during quarantine | 1.36  (.41-3.08) | .18  (.09-.87) | .05  (.04-.75) | .02  (.03-.73) | .02  (.02-.75) | .10  (.04-.94) | .06  (.04-.76) |
| Relatives living out | .83  (.13-2.11) | 1.58  (.59-3.08) | 1.18  (.27-2.99) | .16  (.01-1.03) | .02  (0-.58) | .89  (.12-2.37) | .60  (.06-1.87) |
| Previous Pathologies | .24  (.02-1.15) | .26  (.01-.99) | 1.17  (.29-2.69) | .69  (.07-2.14) | .43  (.01-1.57) | .01  (0-.45) | .02  (.01-.49) |
| Perceived Knowledge | .02  (.01-.45) | .17  (.02-.84) | .36  (.02-1.39) | .13  (.02-.88) | .18  (.02-1.13) | .12  (.01-.79) | .04  (.02-.56) |
| News Seeking | .13  (.04-.76) | .55  (.12-1.52) | .05  (.03-.59) | .48  (.03-1.52) | .42  (.03-1.54) | .07  (.03-.64) | .32  (.05-1.31) |
| Perceived Control | 1.99  (.73-3.8) | .08  (.02-.67) | 1.68  (.47-3.29) | .03  (.01-.55) | .09  (.02-.7) | .01  (.01-.49) | .01  (0-.43) |
| Perceived Efficacy | .10  (.06-.62) | 1.07  (.33-2.33) | .06  (.02-.58) | 1.68  (.56-3.53) | 2.23  (.79-4.26) | .98  (.14-2.47) | .48  (.04-1.53) |
| Negative Emotions | .47  (.14-1.33) | 11.53  (9.33-13.82) | 3.62  (2.3-5.4) | 2.27  (1.32-3.79) | .72  (.36-1.65) | 3.93  (2.57-5.86) | 4.86  (3.53-6.54) |
| Anxiety | .50  (.12-1.49) | 12.24  (9.96-14.81) | 5.21  (3.32-7.51) | 2.63  (1.54-4.36) | 1.11  (.5-2.27) | 4.15  (2.73-5.98) | 6.66  (4.8-8.85) |
| Uncertainty | .28  (.06-1.09) | 6.64  (5.31-8.28) | 1.94  (1.25-3.01) | 3.54  (2.05-5.69) | 2.12  (.89-3.91) | 4.46  (2.89-6.59) | 7.06  (4.99-9.53) |
| Positive Emotions | 3.25  (1.50-5.58) | 1.59  (.60-2.88) | .01  (.01-.48) | .07  (.03-.66) | .09  (.03-.68) | .22  (.03-1.08) | .11  (.02-.76) |
